# Docosahexaenoic acid–mediated, targeted and sustained brain delivery of curcumin microemulsion

**DOI:** 10.1080/10717544.2016.1233593

**Published:** 2017-02-03

**Authors:** Rajshree L. Shinde, Padma V. Devarajan

**Affiliations:** Department of Pharmaceutical Sciences and Technology, Institute of Chemical Technology, Matunga, India Mumbai

**Keywords:** Brain targeting, curcumin microemulsion, docosahexaenoic acid, U-87 MG human glioblastoma cell line, intranasal, intravenous

## Abstract

We disclose microemulsions (ME) of curcumin (CUR) with docosahexaenoic acid (DHA)-rich oil (CUR DHA ME) for targeted delivery to the brain. MEs of CUR (5 mg/mL) with and without DHA-rich oil (CUR Capmul ME) suitable for intravenous and intranasal administration exhibited negative zeta potential, globule size  <20 nm and good stability. Following intravenous delivery MEs exhibited high brain concentration with CUR DHA ME exhibiting a 2.8-fold higher *C*_max_ than CUR solution. Furthermore, high and sustained concentration was demonstrated even at 24 h, which was 8- and 2-fold higher than CUR solution and CUR Capmul ME, respectively. Brain concentrations following intranasal administration were, however, substantially higher as evident from higher *C*_max_ and AUC and sustained compared to corresponding intravenous formulations signifying nose to brain targeting. The high brain concentration of CUR DHA ME is ascribed to the targeting efficiency enabled by DHA-mediated transport across the blood–brain barrier (BBB). Histopathological and nasal toxicity confirmed safety of the MEs. Concentration-dependent cytotoxicity *in vitro*, on human glioblastoma U-87MG cell line was observed with CUR DHA MEs and with the blank DHA ME, implying anticancer potential of DHA. The dramatically low IC_50_ value of CUR DHA ME (3.755 ± 0.24 ng/mL) is therefore attributed to the synergistic effect of CUR and DHA in the ME. The CUR concentration achieved with CUR DHA ME at 24  h which translated to  >66-fold(intranasal) and  >21–fold (intravenous) the IC_50_ value in the U-87MG cell line suggests great promise of CUR DHA ME for therapy of brain cancer by both routes.

## Introduction

Brain disorders are of significant social and economic concern. Neurological disorders, cerebrovascular diseases, brain infections and inflammatory disorders including brain cancer afflict about 1.5 billion people globally. Overcoming the blood–brain barrier (BBB) to enable therapeutic drug concentration in the brain presents the most formidable challenge in effective therapy of such afflictions (Schouten et al., 2002; Devarajan & Shinde, 2011). Intranasal administration provides a noninvasive approach to bypass of the formidable BBB (Laquintana et al., 2009; Shinde et al., 2011).

Enhanced brain uptake of curcumin (CUR) with increased cytotoxicity in astrocytoma–glioblastoma cell line (U-373MG) was demonstrated from a nanostructured lipid carrier (Madane & Mahajan, 2016). Intranasal CUR-loaded PNIPAM nanoparticles showed enhanced bioavailability of curcuminoids in brain as compared to intravenous administration (Ahmad et al., 2014). Intranasal administration of thermosensitive poloxamer hydrogel revealed enhanced CUR targeting to various parts of the brain including the cerebrum, cerebellum, hippocampus and olfactory bulb compared to the intravenous injection (Chen et al., 2013). High targeting efficiency with significantly higher brain/blood ratio was observed from an intranasal microemulsion based *in situ* ion-sensitive gelling system of CUR compared to intravenous administration (Wang et. al., 2012).

Microemulsions (ME) are promising delivery systems for intranasal delivery with manifold advantages of ease of preparation, high loading of hydrophobic and hydrophilic drugs, feasibility of sterilization and ease of administration. MEs for enhanced nose to brain delivery are reported for a number of drugs (Zhang et al., 2004; Vyas et al, 2005, 2006a, 2006b; Jogani et al., 2008), especially hydrophobic drugs. ME components can play a critical role in enhanced brain uptake (Devarajan & Shinde, 2011; Shinde et al. 2011). Mucoadhesive MEs could further enhance nose to brain targeting through improved residence time (Vyas et al., 2005, 2006a, 2006b; Jogani et al., 2008). The high brain delivery of saquinavir, an anti-HIV drug following oral administration of an ME containing flaxseed oil was attributed to docosahexaenoic acid (DHA)-mediated bypass of the BBB (Vyas et al., 2008). Moreover, DHA as a brain nutrient could provide additional advantage. CUR a lipophilic drug presents itself as an attractive candidate for the development of ME while exploiting the bioenhancement feature of the ME. Although the antitumor, anti-inflammatory, antiamyloid, anti-ischemic, antioxidant properties of CUR have been extensively reported, poor solubility proves a major limitation factor in therapeutic exploitation (Aggarwal & Shishodia, 2006; Anand et al., 2007), major therapeutic properties of CUR. Delivering CUR effectively could enable manifold therapeutic applications, while enhanced brain delivery could open up improved therapeutics of brain afflictions.

The aim of the present study was to design a stable ME of CUR with DHA and to determine the role of DHA in delivering effective concentrations of CUR to the brain by intravenous and intranasal route. The objectives of the study were to compare enhanced brain delivery by intranasal and intravenous route. Yet another objective was to evaluate the cytotoxicity of the CUR DHA ME in a brain cancer glioblastoma cell line as an indicator of anticancer efficacy.

## Materials and methods

### Materials

Curcumin was obtained as a gift sample from Konark herbals and Healthcare, India (assay 99.9%). DHA rich oil (Croda Chemicals (India) Private Limited), Capmul MCM (Indchem International, Abitec Corporation India) were obtained as a gift samples. Tween 80, ethanol, propylene glycol, *N,N*-dimethylacetamide, methanol, acetonitrile were procure from Merck India Pvt. Ltd. All other chemicals were of analytical reagent grade or HPLC grade.

### Cell culture study

The cell lines U-87 MG was procured from National Center For Cell Science (NCCS), Pune, India. Cells were cultured separately in modified Dulbecco’s medium (Invitrogen, Carlsbad, CA) with 10% fetal bovine serum (FBS) (culture media). Culture media were supplemented with 100 U/mL penicillin and 100 μg/mL streptomycin (Gibco). The *in vitro* assays were carried out in 96-well cell culture plates (Corning).

### Drug-loaded microemulsions

The ME composition described in our earlier study, comprising CUR 5 mg/mL was developed (Shinde et al., 2015). Briefly, CUR (5 mg) was added to the preformed ME (1 mL) comprising 60% Tween80:ethanol (3:1), 10% [DHA + Capmul MCM(1:1)] and 30% water by weight in Eppendorf tubes (1.5 mL) and vortexed on a cylcomixer. MEs were prepared with and without DHA-rich oil to evaluate role of DHA on brain uptake. The oil concentration was maintained at 10% and comprised either DHA-rich oil:Capmul MCM (1:1) referred to as CUR DHA ME or with Capmul MCM as oil are referred to as CUR Capmul ME.

### Characterization

#### Drug content

CUR MEs were suitably diluted with methanol and monitored for drug content by HPLC at 425 nm (CUR).

#### Globule size and zeta potential measurement

The average globule size, polydispersity index [PDI] and zeta potential of MEs was determined using Zetasizer [ZS 90, Malvern, UK] at 25 °C after appropriate dilution with double-distilled water (1:50) (Patel et al., 2010).

#### Small-angle neutron scattering (SANS)

Small-angle neutron scattering (SANS) of the ME was recorded using a SANS diffractometer at the Bhabha Atomic Research Center, Trombay. CUR-loaded ME was diluted with deuterium oxide (ME: D_2_O; 1:10 v/v) and analyzed by using beryllium oxide filtered beam of a wavelength of 5.2 Å. Samples were held in a quartz sample holder of 0.5 cm thickness, at a temperature of 30 °C (Jain et al., 2010).

#### Transmission electron microscopy (TEM)

TEM analyses were performed on a FEI Tecnai 12 BT instrument operated at 120 kV. CUR DHA ME was diluted with Milli-Q water at a ratio of 1:50 and mixed by vortexing on cylcomixer. A drop of the ME was placed on a TEM grid covered by a holey carbon film, stained with 2% uranyl acetate for 10 min and blotted with filter paper to form a thin liquid film on the grid. TEM micrographs were recorded using Image Analysis software and CCD camera (Setthacheewakul et al., 2010).

#### Viscosity

The viscosity of MEs was evaluated on a cone and plate programable rheometer (Physica MCR101, Anton Paar, Germany) connected to a digital thermostatically controlled circulating water bath (Polyscience, Model 9101) having spindle CP35-2-SN20784 (d = 0.147 mm) (Pastoriza-Gallego et al., 2011). Twenty-five measurements between the shear rates of 0.01 to 100 s^−^ ^1^ were taken after the equipment and sample was equilibrated for 5 min following loading at a constant temperature of 20 °C. The RHEOPLUS/32 V3.40 software was utilizes for data analysis, three replicates were recorded and the average value determined.

#### Mucoadhesion test

Mucoadhesion of the ME formulations were performed by using CT3 Texture analyzer (100 g). The test was conducted in the compression mode with a trigger load of 0.5 g. An aqueous solution of 3% w/v porcine (100 μL) mucin was applied on the tissue holder and allowed to dry. This served as the mucoadhesive substrate. Cylindrical probe with closed bottom (TA-10) was dipped into the ME containing a watchglass which results in formation of thin and continuous film on the surface of probe. The probe was lowered to just touched the mucoadhesive substrate, was allowed contact for 10 sec and withdrawn at a speed of 0.5 mm/sec. Distilled water served as reference. Data analyzed using TexturePro CT V1.3 Build 15 software (Thirawong et al., 2007).

#### Fourier transform infrared spectroscopy (FTIR)

FTIR for CUR drug powder and CUR DHA ME were recorded on a Perkin-Elmer FTIR spectrophotometer by the potassium bromide (KBr) disk method from 4000 to 500 cm−^1^. Samples were crushed to a fine powder, mulled with anhydrous potassium bromide, compressed to form a thin transparent pellet and subjected to FTIR.

### Stability evaluation

#### Freeze-thaw cycle

MEs were subjected to four cycles of heating (40 °C) and cooling cycles at 40 °C and freezing (0 °C) temperature with storage at each temperature for not less than 48 h.

#### Accelerated stability studies

CUR MEs were filled in stoppered glass vials and were subjected to accelerated stability studies as per International Conference on Harmonization (ICH) guidelines at 30 °C ±2 °C/65% relative humidity (RH), and 40 °C ± 2 °C/75% RH. Samples were withdrawn at specific time intervals and evaluated for globule size, appearance and drug content by HPLC.

### Hemolysis and serum stability

*In vitro* hemolytic study was carried out using a reported method (Bock & Müller, 1994). Red blood cell (RBC) suspended in the 0.9% w/v NaCl solution served as negative control and distilled water serve as positive control.

To 10% rat plasma in phosphate-buffered saline (PBS) pH 7.4, freshly prepared CUR DHA ME was added in a ratio of 1:1 in an Eppendorf tube and incubated at 37 ± 0.5 °C. Aliquots (0.1 mL) were withdrawn up to 6 h. Globule size was monitored by Zetasizer Nano ZS (Malvern Instruments Ltd., UK) (Han et al., 2006).

### *In vivo* evaluation

#### Animals

All animal experiments in this study were performed in compliance with the Protocols of Animal Use and Care and was approved by the Institutional Animal Ethics committee [Protocol No. ICT/IAEC/2012/P16 (Pharmacokinetic and brain uptake study); Protocol No. ICT/IAEC/2013/P66 (Toxicity Study)]. Prior to commencement, rats were acclimatized for one week at controlled temperature of 22 ± 1 °C with relative humidity of 60–70% in polypropylene cages. Rats were fed a standard diet (Amrut brand, Sangli, India) and provided water *ad libitum*. Sprague-Dawley (SD) rats (4–5 months) weighing 200–250 g were selected.

#### Drug administration

Rats were fasted for 12–16 h before the study with free access to water. The study was conducted in six groups of rats (*n* = 4), three groups for intranasal and three groups for intravenous administration. Prior to administration, the rats were partially anesthetized by exposure to diethyl ether. CUR DHA ME, CUR Capmul ME and CUR solution were administered intranasally and intravenously to rats. CUR solution was prepared dissolving CUR (5 mg/mL) in a solvent system comprising propylene glycol: *N,N*-dimethylacetamide (95:5). **Intravenous:** MEs were diluted prior to administration. 50 μL equivalent to 250 μg CUR was diluted to 0.2 mL with PBS to facilitate ease of injection and diluted MEs at dose of 1 mg/kg administered through the tail vein. **Intranasal:** Undiluted MEs were directly instilled into the nasal passage. Rats were held by the neck with head tilted backwards during intranasal administration and formulations (50 μL) were instilled into the rat nostril with the help of a micropipette. The same dose of CUR (1 mg/kg) was administered by both routes.

#### Drug extraction

The deproteination method using methanol was utilized to extract CUR from plasma/brain homogenates. Brain homogenates were obtained by homogenization of brain samples in PBS: Acetonitrile (ACN) (50:50) followed by centrifugation at 10 000×*g* (10 min) to obtain supernatants. The supernatant (200 μL) was mixed with methanol 300 μL in 1.5-mL eppendorf tube and vortexed, followed by addition of 500 μL of 2% acetic acid (pH 3): ACN (50:50) and vortexing for 3 min. The supernatant obtained by centrifugation at 10 000×*g* (10 min) was quantified for CUR by a validated HPLC method.

Reverse-phase high-performance liquid chromatography (RP HPLC) method used to distinguish between CUR, its analogs and its degradation products to enable quantification of CUR was developed. The analysis of CUR was carried out using a HPLC system (Jasco PDA), having 100 μL injection loop with C18 column Waters Spherisorb® 5 μm ODS2 (4.6 × 250 mm). Analytical column equipped with solvent delivery pump and Jasco MD-2010 multiwavelength detector operated at 425 nm. The mobile phase composed of 2% glacial acetic acid (pH3) and ACN in a ratio of 50:50, pumped at a flow rate of 0.9 mL/min with retention time of 11.55 ± 0.28, 12.95 ± 0.36 and 13.28 ± 0.25 min, respectively, for bisdemethoxycurcumin, demethoxycurcumin and curcumin.

#### Pharmacokinetic study

Blood (500 μL) was collected from the retro-orbital plexus into heparinized tubes at various time points 0.083, 0.5, 1, 6 and 24 h after dosing. Plasma was separated by centrifuging for 5 min at 5000 ×*g* and stored at −70 °C until HPLC analysis. The extraction of CUR was carried out as detailed under the drug extraction and the supernatant analyzed by the validated HPLC method as reported in the following sections of drug extraction.

#### Brain uptake study

For the brain uptake study, rats for the pharmacokinetic study were euthanized at 0.083, 0.5, 6 and 24 h by exposure to excess CO_2_. Subsequently, the brains were isolated, washed twice using normal saline solution and weighed. Samples were stored at −70 °C prior to HPLC analysis. Drug extraction is detailed under the drug extraction section. Supernatant was analyzed by HPLC detailed in the following sections of the drug extraction.

Brain tissues of 5 μm thickness were sectioned using a freezing cryostat (Microm HM 520) and fixed in 10% formaldehyde for 10–15 min. The brain sections were observed under a fluorescence microscope (Olympus IX-71) using a green filter, which relied on the auto fluorescence of CUR for visualization.

#### Targeting efficiency by intranasal administration

Drug-targeting efficiency (DTE) and direct transport percentage (DTP) indexes were implemented (Kozlovskaya et al., 2014), DTE was calculated using the following equation:

(1) % DTE=(Bi.n./Pi.n.)/(Bi.v./Pi.v.)×100

where Bi.n. = AUC _0–24_ [brain] following intranasal administration,

Pi.n. = AUC _0–24_ [Plasma] following intranasal administration,

Bi.v. = AUC _0–24_ [brain] following intravenous administration,

Pi.v. = AUC _0–24_ [Plasma] following intravenous administration,

In order to elucidate nose to brain direct transport more clearly, direct transport percentage (DTP) was calculated using following equation:

(2) DTP [%]=Bi.n.-Bx/Bi.n.×100

where Bx is the brain AUC fraction contributed by systemic circulation through the BBB following intranasal administration and

(3) Bx=[Bi.v./Pi.v.]×Pi.n.

#### Subacute toxicity and nasal toxicity

Male SD rats (*n* = 8) were divided in to six groups. Group I, II and III were administered formulations intravenously through the tail vein, while IV, V, VI administered formulation intranasally as described below.

Groups were as indicated: group I: CUR DHA ME (1 mg/kg of CUR), Group II: Blank DHA ME, Group III: vehicle control (Saline) and Group IV: CUR DHA ME (1 mg/kg of CUR), Group V: Blank DHA ME, Group VI: positive control (1% sodium deoxycholate w/v) for intranasal. All groups were administered daily intravenous dose of (0.2 mL) or intranasal dose (50 μL/nostril) for 7 or 14 consecutive days. All animals were monitored for mortality, abnormal breathing and unusual behavior, till 14 days. Blood was collected from the retro orbital plexus on day 7 and day 14 day for hematology and serum biochemistry. Four animals from each group were sacrificed at the end of day 7 and day 14 and organs such as brain, liver, lung, spleen, kidney and heart were isolated, organs were fixed in 10%v/v formalin solution, embedded in wax, microsectioned and stained with hematoxylin and eosin (H and E) and histopathological examination carried out. Nasal tissues were isolated from the groups administered formulations intranasally to evaluate nasal toxicity. The decalcified tissues were embedded in paraffin and sectioned using a microtome (Nikon Fx-35A, Japan) (Dong et al., 2010). Following staining with H and E, the sections were observed under an optical microscope.

### *In vitro* cytotoxicity study in U-87 mg human glioblastoma cells line

Cytotoxicity was determined by the MTT [3-(4, 5-dimethylthiazol-2-yl)-2, 5-diphenyl tetrazolium bromide] assay as previously described (Mulik et al., 2010). The U-87 MG cells were grown on Dulbecco's modified Eagle's medium (DMEM) supplemented with 10% FBS at 37 °C. The cell count was determined using a hemocytometer. Briefly U-87MG cells were seeded in the 96-well plates at a density of 1 × 10^4^ cells/0.1 mL of medium in each well and allowed to adhere by incubating for a period of 24 h at 37 °C. The medium was discarded and replaced with fresh medium (0.1 mL) containing various concentrations of CUR solution, CUR DHA ME, Blank DHA ME, CUR Capmul ME, Blank Capmul ME. The plate was incubated at 37 °C for a period of 1 h. At the end of 1 h, the medium in each well was discarded and 50 μL of MTT solution (5 mg/mL in phosphate-buffered saline) was added to each well and the plate incubated at 37 °C for a period of 4 h. DMSO (100 μL) was then added to each well to dissolve the formazan crystals formed and the plates were read immediately at 540 nm on a microplate reader (Thermo Fisher Scientific, Finland). The percentage cell viability was calculated was calculated using the formula:

$$\mathrm{Cell}\;\mathrm{viability}=\left[A_{\mathrm{sample}}/A_{\mathrm{control}}\right]\times 100$$

The half maximal inhibitory concentration (IC_50_) calculated by using GraphPad Prism 5 software (Mathew et al., 2012; Mukerjee & Vishwanatha, 2009).

### Statistical analysis

All data are expressed as mean ± SD. For multiple-group comparison, one-way analysis of variance (ANOVA) was used followed by Dunnett's tests (GraphPad Prism 5 software). Specific comparison between groups was carried out using the unpaired Student’s *t*-test (two tailed). All data were dose and weight normalized. Pharmacokinetic parameters for formulations were calculated using Basica software. Differences were considered statistically significant at *p* < 0.05.

## Results and discussion

Treatment of brain disorders including brain cancer is a challenged by the tightly regulated BBB and its unique ability to protect the brain from xenobiotics. Conventional therapeutics, although effective, remain critically below levels of optimum therapeutic efficacy. DHA is important for brain health and furthermore is reported for antitumor efficacy (Nasrollahzadeh et al., 2008). The presence of specific fatty acid DHA transporters in BBB presents the possibility of rapid and high brain uptake mediated by DHA in the ME. ME with DHA-rich oil therefore could be a useful vehicle for the solubilization, enhanced bioavailability and enhanced drug delivery to the brain of poorly soluble drugs such as CUR. Intranasal delivery is a promising route for enhanced brain targeting, nevertheless CUR for anticancer therapy may necessitate administration of large doses of drug not be feasible through intranasal route. Hence, we evaluated CUR ME by intravenous and intranasal route for targeted brain delivery. All excipients in the ME were safe for intravenous and intranasal administration to ensure that the same ME could be administered by both routes.

### Preparation and characterization of microemulsions

#### Drug-loaded microemulsions

**Figure 1.**
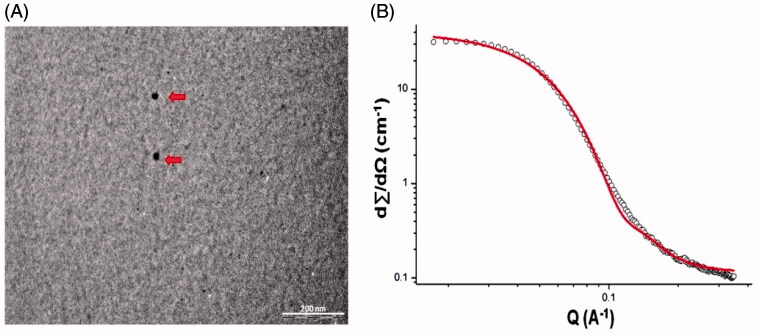
Characterization of CUR DHA ME: TEM micrograph of CUR DHA ME (A) and SANS Image of CUR DHA ME (B).

The MEs were stable on dilution (50X and 100X) irrespective of oil [Capmul ME/DHA-rich oil: Capmul (1:1)] in the MEs with average globule size  < 20 nm and negative zeta potential (−8.64 ± 0.87 to −10.54 ± 0.63). The small globule size and low polydispersity indices (0.217 ± 0.051 to 0.243 ± 0.03) for MEs indicated large surface area with the potential of high drug permeability. Stability was confirmed by the negative zeta potential (Vyas et al., 2005, 2006b; Kumar et al. 2008a, 2008b). The pH of all the ME formulations was found to be in range of 5.4–5.5 and considered suitable for intranasal (Washington et al., 2000) and intravenous administration. TEM (Figure 1A) micrograph of ME revealed spherical shape of the droplets and a size < 20 nm. SANS is proven as a unique and powerful tool in elucidating the structure, interaction and phase transitions in lyophilic colloids especially in micellar and microemulsion system. SANS analysis confirmed the existence of spheres with a radius of 36 Å (3.6 nm) and a polydispersity index of 0.2 (Figure 1B) and no other surfactant mesophases. However dilution of MEs was essential to enable intravenous administration. Dilution of the MEs 1:100, 1:100 revealed no change in globule size (*p * < 0.05). Drug content for CUR Capmul ME (98.87 ± 2.16%) and CUR DHA ME (99.14 ± 2.08) found to be > 95%. Viscosities of the CUR Capmul ME (168 ± 2.13 cp) and CUR DHA ME (171 ± 1.92 cp) were comparable and did not change with change of the oil phase and were considered suitable for intranasal administration.

##### Fourier transform infrared spectroscopy (FTIR)

The FTIR spectrum. of CUR and CUR DHA ME revealed all the characteristic frequencies and vibrational assignments of CUR as seen by the O-H stretching at 3437 cm^−1^, C-H stretching absorption bands at 2856 and 2924 cm^−1^, an enol carbonyl stretching absorption band at 1622 cm^−^ ^1^,-C-H bending absorption band at 1456 cm^−1^ and C-O stretching absorption band at 1126 cm^−^ ^1^ (Supplementary Figure S1). These peaks are in accordance with the structure and functional groups of CUR confirming the chemical stability of the drug and no chemical interaction between drug and excipient in the MEs.

### Stability evaluation

CUR MEs subjected to freeze thaw cycling exhibited no change in color, no precipitation or change in globule size on dilution. CUR content of  >95% for CUR was evident at the end of 6 months even after exposure to 40 °C/75% RH as per ICH guidelines, confirming good stability.

### Mucoadhesion

The undiluted ME exhibited good mucoadhesion of 105.2 ± 2.16 g and 109.26 ± 3.42 g for CUR Capmul ME and CUR DHA ME, respectively. This mucoadhesive property although surprising could provide an important advantage of nasal mucoretention to deliver sustained drug concentration to the brain. Nevertheless, such mucoadhesion was not evident on dilution, conferring the MEs suitable for intravenous administration.

### Hemolysis and serum stability

Hemolysis of < 10% and no change in globule size (*p* > 0.05) over 6 h in the serum stability study confirmed safety of the MEs for intravenous administration (Dobrovolskaia et al., 2008).

### *In vivo* evaluation

The high concentration of DHA in the brain capillary endothelium suggests that DHA is taken up from the diet via blood plasma, by DHA transporters including specific fatty acid-binding lipoprotein carriers (Lukiw & Bazan, 2008). DHA therefore presents the possibility of receptor-mediated endocytosis (RME) to overcome BBB (Chen et al., 2009). ME containing oils rich in DHA could provide the added advantage of enhanced drug delivery to brain. The antitumor property of DHA could provide additional advantage in the CUR ME designed for anticancer activity (Tiwari et al., 2006; Ganta & Amiji, 2009; Siddiqui et al. 2013a, 2013b).

#### Drug extraction

CUR extraction from plasma and brain homogenate was consistent and reproducible. Extraction efficiency was optimized and recovery of extracted sample from plasma and brain homogenate was found to be greater than 90%, when analyzed by the HPLC method reported earlier (Shinde et al., 2015).

#### Plasma and brain pharmacokinetics following intravenous administration

**Figure 2.**
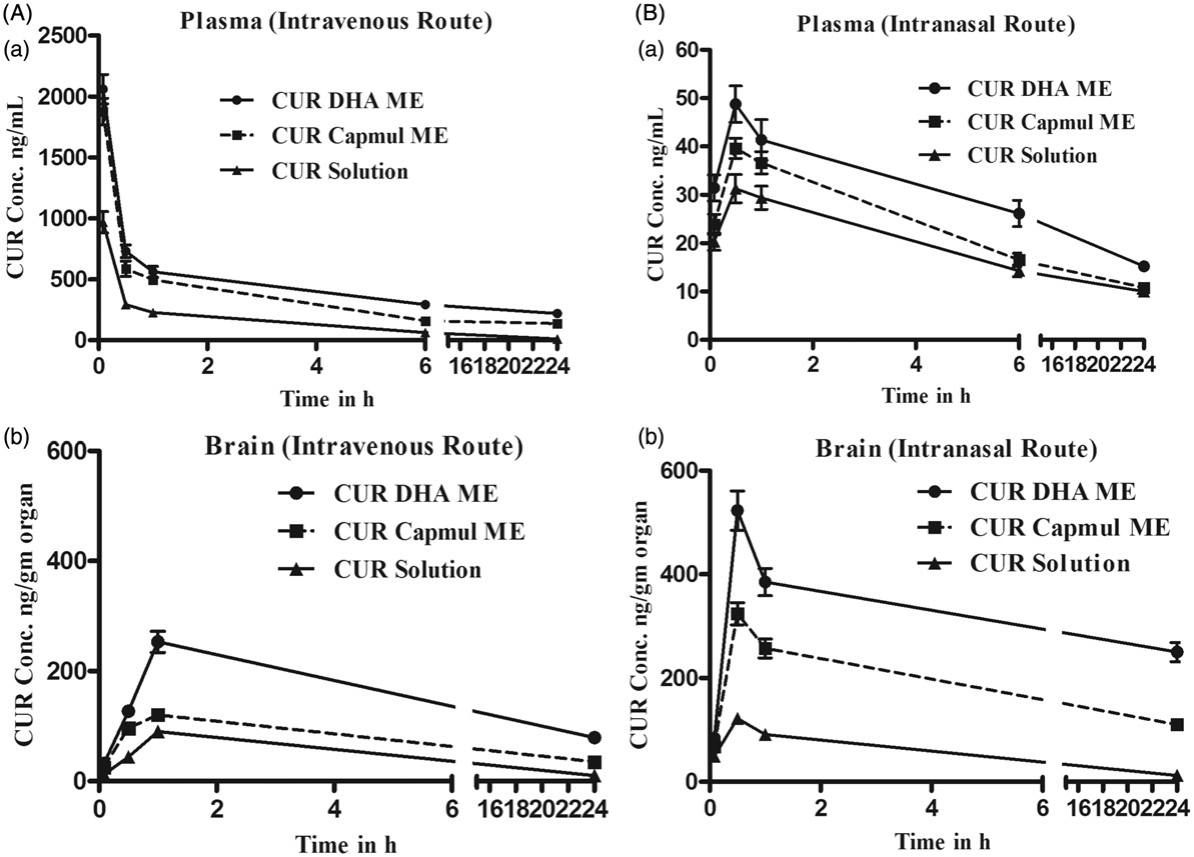
(A) Intravenous CUR concentration vs. time profiles for plasma (a) and brain (b) following intravenous administration of the CUR in MEs or CUR solution to SD rats. (B) Intranasal CUR concentration versus time profiles plasma (a) and brain CUR (b) following intranasal administration of the CUR in MEs or CUR solution to SD rats.

Plasma and brain pharmacokinetics of CUR following intravenous administration are depicted in Figure 2(A). High plasma levels and slower elimination was demonstrated by MEs following intravenous administration compared to CUR solution. The oil in the MEs influenced the *in vivo* behavior with CUR DHA ME exhibiting significantly higher *C*_max_ (maximum concentration), higher area under the curve (AUC) and longer half-life (*t*_1/2_) compared to CUR Capmul ME (Table 1). This is attributed to DHA being a long-chain triglyceride, hence exhibiting slow metabolism (Tamilvanan, 2009; Picq et al., 2010; Siddiqui et al., 2013b). The enhancement in plasma AUC compared to CUR solution was 3.79-fold for CUR Capmul ME and a striking 5.74-fold for CUR DHA ME (Figure 2A (a)). Brain concentrations following intravenous administration also exhibited similar trends with CUR DHA ME showing maximum *C*_max_, AUC and *t*_1/2_. Bioenhancement exhibited by CUR DHA ME with reference to CUR solution in the brain was 2.89-fold and lower with CUR Capmul ME 1.75-fold (Figure 2A (b)). Importantly, following intravenous administration the *C*_max_ in the brain with CUR Capmul ME was barely 1.3-fold compared to CUR solution. Inclusion of DHA enable a marked increase in 2.8-fold confirming the positive role of DHA in enhancing the brain-targeting efficiency by intravenous route. Even at 24 h significant concentration of CUR were detected in the brain with CUR DHA ME showing an almost 8-fold higher CUR concentration compared to CUR solution and 2-fold higher compared to CUR Capmul ME (Table 1). This high uptake is attributed to the ability of DHA to traverse the BBB facilitated by the natural endogenous transporters, major facilitator superfamily domain-containing protein 2a (Mfsd2a) (Nguyen et al., 2014), assisted by lipoprotein-mediated endocytotic uptake across the BBB facilitated by tween 80 (Kreuter et al., 2003; Goppert & Müller, 2005).

**Table 1.** Plasma and brain pharmacokinetics parameters upon intravenous administration of the MEs of CUR or CUR solution in SD rats.

Parameters	Intravenous (Plasma)			Intravenous (Brain)		
CUR	CUR Solution	CUR Capmul ME	CUR DHA ME	CUR solution	CUR Capmul ME	CUR DHA ME
*C*_max_ (ng/mL)	968.11 ± 67.1	1875.45 ± 91.6	2059.8 ± 103.6	90 ± 6.82	120 ± 8.61	253 ± 18.74
*t*_max_ (h)	0.0833	0.0833	0.0833	1	1	1
Kel (h^−1^)	0.1241 ± 0.0091	0.0440 ± 0.0032	0.0334 ± 0.0022	0.0785 ± 0.0053	0.048 ± 0.0027	0.0347 ± 0.0016
*t*_1/2_ (h)	5.582 ± 0.42	15.74 ± 0.86	20.324 ± 1.3	8.817 ± 0.92	14.42 ± 0.97	19.94 ± 1.35
AUC_0-24_	1322.411 ± 41.54	5020.32 ± 88.73	7602.64 ± 117.25	1078.17 ± 34.93	1931.76 ± 42.71	3124.98 ± 48.28
Conc at 24 h	10.62 ± 1.2	135.82 ± 12.8	220.49 ± 21	10 ± 8.3	35 ± 2.1	79 ± 6.3
Relative bioavailability (%)^a^				–	179.17	289.84

^a^Intravenous CUR solution as reference.

#### Plasma and brain pharmacokinetics following intranasal administration

Following intranasal administration, plasma concentrations were significantly low compared to intravenous (Figure 2B (a)), although the *C*_max_, AUC, *t*_1/2_ were in the order CUR solution < CUR Capmul ME < CUR DHA ME (Table 2). However, brain concentrations following intranasal administration were strikingly higher compared to intravenous specially with the MEs (Figure 2A (b) and 2B (b)). The comparative enhancement in *C*_max_ following intravenous and intranasal was CUR solution 1.3, Capmul ME 2.7-fold, CUR DHA ME 2.06-fold. An early brain *t*_max_ following intranasal administration coupled with an enhanced *t*_1/2_ particularly with the CUR DHA ME proposed a major advantage of DHA in ME. CUR solution exhibited lower *t*_1/2_ and comparable AUC in the brain by both intravenous and intranasal routes, while the MEs revealed significant enhancement in AUC by intranasal route which was markedly higher for CUR DHA ME (Figure 2B (b)). The lower *t*_max_ values for the brain (0.5 h) following intranasal administration compared to intravenous administration *t*_max_ values for the brain (1 h) is attributed to preferential nose to brain transport following intranasal administration enabling rapid uptake. DTE(%) values were in order CUR solution (433.349%) < CUR Capmul ME (1209.86%) < CUR DHA ME (**1615.429%**) with % enhancement of 2.7 and 3.7 for CUR Capmul ME and CUR DHA ME, respectively, compared to CUR solution. Similarly, DTP (%) values were in order CUR solution (76.924%) < CUR Capmul ME (96.10%) < CUR DHA ME (**96.88%**). Nose to brain targeting was confirmed by the high CUR levels for the MEs seen coupled with the high DTE and DTP values that were significantly greater than CUR solution.

**Table 2.** Plasma and brain pharmacokinetics parameters upon intranasal administration of the MEs of CUR or CUR solution in SD rats.

Parameters	Intranasal (Plasma)			Intranasal (Brain)		
CUR	CUR solution	CUR Capmul ME	CUR DHA ME	CUR solution	CUR Capmul ME	CUR DHA ME
*C*_max_ (ng/mL)	31.25 ± 2.8	39.62 ± 2.3	48.75 ± 3.1	122 ± 7.89	324 ± 22.43	523 ± 30.95
*t*_max_ (h)	0.5	0.5	0.5	0.5	0.5	0.5
Kel (h^−1^)	0.1430 ± 0.013	0.0481 ± 0.0029	0.0399 ± 0.0021	0.0936 ± 0.0068	0.04162 ± 0.0026	0.0253 ± 0.0015
*t*_1/2_ (h)	10.12 ± 0.78	14.4 ± 0.83	17.35 ± 0.81	7.4 ± 0.74	16.64 ± 1.24	27.32 ± 1.38
AUC_0-24_	357.64 ± 23.4	451.13 ± 34.21	581.56 ± 41.11	961.4184 ± 26.46	4450.30 ± 43.87	6739.546 ± 73.45
Conc. At 24 h	10 ± 1.1	10.75 ± 1.2	15.21 ± 1.1	12 ± 1.3	110 ± 9.3	250 ± 18.45
Relative bioavailability (%)^a^				89.171	412.76	625.091

^a^Intravenous CUR solution as reference.

Fluorescence images of brain tissue sections that showed significant yellow fluorescence confirming CUR brain uptake, while the intensity of the yellow fluorescence established the superiority of the CUR DHA ME in enhancing brain uptake Supplementary Figure S2.

Superiority of the CUR DHA ME confirmed the role of DHA in enhanced brain targeting by both intravenous and intranasal route. Although the levels following intranasal administration were significantly higher than intravenous, the levels in the brain even by intravenous administration were significant, suggesting both intravenous and intranasal routes as promising.

#### Subacute toxicity and nasal toxicity

Serum biochemistry and hematology data were comparable with the vehicle control even at the end of 14 days. Histopathology of all vital organs including brain revealed no significant changes at the end of 14 days. Supplementary Figure S3

**Figure 3.**
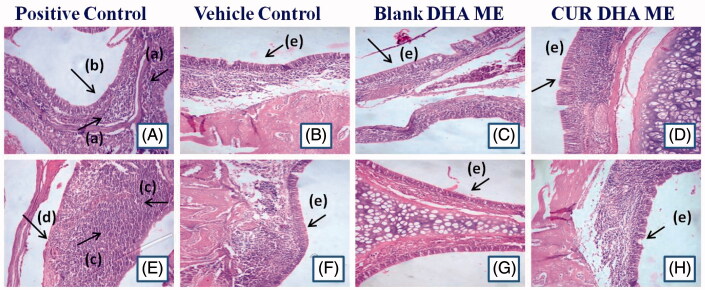
Photomicrographs of positive control [1% sodium deoxycolate solution, (A, 7 days) no cilia on the mucosa was observed (E, 14 days)], Vehicle control* [saline, (B, 7 days) (F, 14 days)], Blank DHA ME* [(C, 7 days) (G, 14 days)] and CUR DHA ME * [(D, 7 days) (H, 14 days)] (10 × 40 magnification, *n* = 4). *(a) Moderate infiltration, (b) Degenerative changes in nasal epithelium, (c) Severe leucocytic infiltration, (d) Extensive desquamation of nasal epithelium, (e) Intact nasal epithelium with healthy appearance.

The nasal toxicity study also showed no changes. Figure 3 clearly depicts the changes in the nasal epithelium following administration of the ME formulations and the positive control and vehicle control. The positive control exhibited mild to moderate with the desquamation of epithelium that was multifocal and leucocytic exudates indicative of minimal inflammatory reaction on day 7. On day 14, the reaction was exacerbated resulting in severe hyperemia, desquamation of epithelium to a moderate degree with neutrophilic exudates suggesting enhanced inflammatory reaction. No such changes were evident with the ME formulations even at the end of 14 days, reassuring the safety of the CUR MEs.

### *In vitro* cytotoxicity study in U-87 MG human glioblastoma cells line

Blank MEs were prepared corresponding to equivalent dilutions of CUR MEs. Blank Capmul ME revealed marginal cytotoxicity at lower dilution. The substantial increase in cytotoxicity at lower dilutions were attributed to possible surfactant based cytotoxicity. At lower concentrations up to 50 ng/mL, significantly higher cytotoxicity seen with the blank DHA ME compared to blank Capmul ME Supplementary Figure S4. Further the greater cytotoxicity exhibited by the blank DHA MEs compared to CUR solution confirmed the anticancer property of DHA reported against glioblastoma cell line. Interestingly among the CUR MEs the CUR DHA ME revealed significantly higher cytotoxicity compared to the CUR Capmul ME ascribed to a possible synergy of DHA with CUR (Siddiqui et al., 2013a) Supplementary Figure S4. Such synergistic behavior of DHA with arsenic acid in number of cell lines (Baumgartner et al., 2004) and with paclitaxel in BT-474 and SK-BR-3 breast cell lines that have been linked to lipid peroxidation (Menendez et al., 2005) is reported. DHA-mediated enhanced uptake of CUR by modulating plasma membranes in glioblastomas could have also played a critical role (Harvey et al., 2015). Studies have shown that DHA incorporation changed the chemical and physical properties of cell membrane phospholipids to enhance uptake of various anticancer drugs, including vincristine in a drug-resistant neuroblastoma cell line (Ikushima et al., 1990). Furthermore, DHA has also been shown to directly modulate the activities of intracellular mediators involved in cell survival and apoptosis including NFkB, PPARa, MAP kinase, AKT, COX-2, Bcl2 and Bax (Zhuo et al., 2009). *In vivo* studies by Siddiqui et al. demonstrated that a combination of DHA with CUR reduced the incidence of breast tumors, delayed tumor initiation, and reduced progression of tumor growth (Siddiqui et al., 2013a), although signaling mechanism specifically associated with the combination effect have yet to be elucidated. Treatment of prostate cancer cells with DHA induced cell cycle arrest and apoptosis (Berquin et al., 2007) and decreased prostate tumor growth in vivo (Trebelhorn et al., 2014). The anticancer activity of DHA on the U-87 MG glioblastoma cell lines could be due to the natural affinity of DHA to the neuronal cells. In addition, DHA induced lipid peroxidation could have contributed to enable tumor cells destruction (Baumgartner et al., 2004). IC_50_ values depicted in Table 3 confirmed the superiority of CUR DHA ME against the glioblastoma cell line and synergistic effect of DHA with CUR.

**Table 3.** IC50 (ng/mL) and fold enhancement in CUR brain concentration with respect to IC_50_ value.

		Fold Enhancement at *C*_max_		Fold Enhancement at 24h	
Formulation	IC_50_ in ng/mL	Intravenous *C*_max_ at 1 h	Intranasal *C*_max_ at 0.5 h	Intravenous	Intranasal
CUR DHA ME	**3.755**±**0.24**	67.01	138.54	21.038	66.58
CUR Capmul ME	172.3 ± 11.74	–	<2 fold	0.638	–
Blank DHA ME	502.7 ± 24.65	–	–	–	–
Blank Capmul ME	656.1 ± 33.96	–	–	–	–
CUR Solution	747.8 ± 53.7	–	–	–	–

### Brain concentrations and IC_50_ value

Brain concentrations were related to the IC_50_ value and the fold enhancement value is depicted in Table 3. Notably, the enhancement in IC_50_ was minimum 21-fold even at 24 h following intravenous administration of CUR DHA ME and minimum 66-fold following intranasal administration, while no such enhancement was seen with CUR Capmul ME or CUR solution. Levels of CUR achieved with CUR DHA ME, therefore, indicate the possibility of high efficacy of the CUR DHA ME in glioblastoma cell line.

## Conclusion

The superiority of CUR DHA ME in enabling targeted brain delivery of CUR by both intravenous and intranasal administration presents this new formulation as a promising and versatile formulation for application in brain cancer.

## Supplementary Material

Updated_Supplementary_Figures-Devarajan_Shinde__2_.docx

## Notes

**Supplementary material available online**
