# Supplementary material for: Docosahexaenoic acid–mediated, targeted and sustained brain delivery of curcumin microemulsion
Source: Drug Deliv. 2017 Feb 3;24(1):152–61. doi: 10.1080/10717544.2016.1233593 (PMC8244623; doi:10.1080/10717544.2016.1233593)
Supplement: Updated_Supplementary_Figures-Devarajan_Shinde__2_.docx [file IDRD_A_1233593_SM6835.docx]

Supplementary Figures:

**Figure S1**: FTIR Spectrum of CUR (A), CUR DHA ME (B)

**Figure S2:** Fluorescence micrograph of brain sections at 0.5 and 1h

**Figure S3:** Photomicrographs of brain following intravenous administration Vehicle Control (A, 14 days), Blank DHA ME (B, 14 days)] and CUR DHA ME (C, 14 days) and photomicrograph of brain following intranasal administration Vehicle Control (D, 14 days), Blank DHA ME (E, 14 days) and CUR DHA ME (F, 14 days) (10× 10 magnification, n = 4).

**Figure S4:** % Cell Cytotoxicity by MTT Assay at 1 h (mean ± S.D.; n = 3)


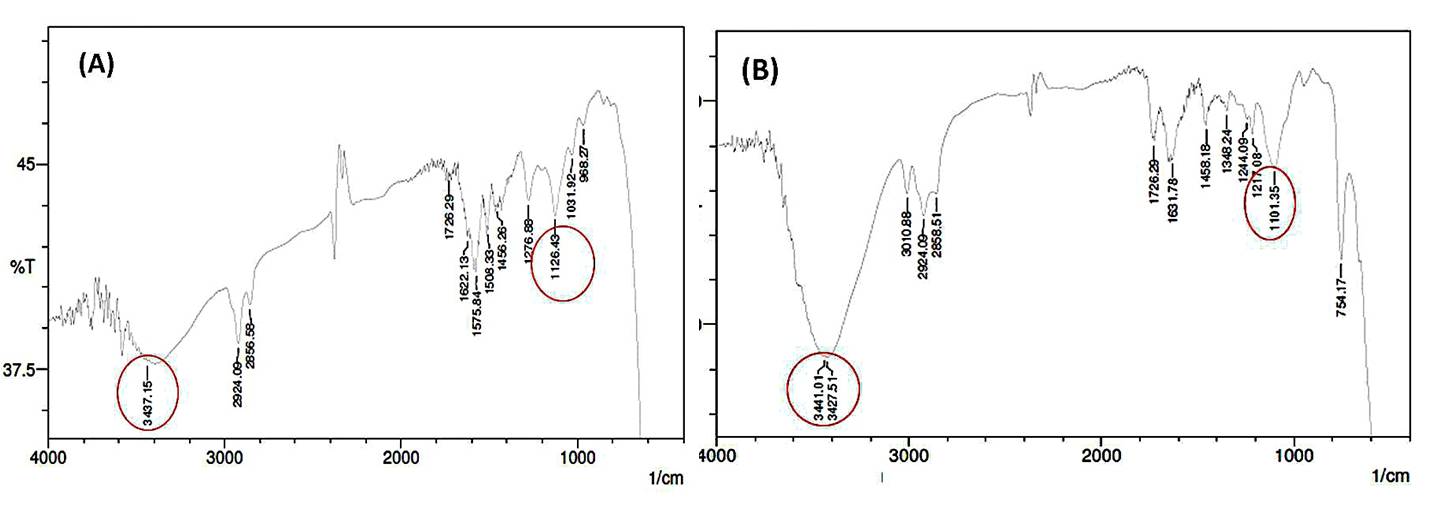


**Figure S1**: FTIR Spectrum of CUR (A), CUR DHA ME (B)

Explanation: The FTIR spectrum. of CUR and CUR DHA ME revealed all the characteristic frequencies and vibrational assignments of CUR as seen by the O-H stretching at 3437 cm^-1^, C-H stretching absorption bands at 2856 and 2924 cm^-1^ , an enol carbonyl stretching absorption band at 1622 cm^-1^,-C-H bending absorption band at 1456 cm^-1^ and C-O stretching absorption band at 1126 cm^-1^. These peaks are in accordance with the structure and functional groups of CUR confirming the chemical stability of the drug and no chemical interaction between drug and excipient in the MEs.


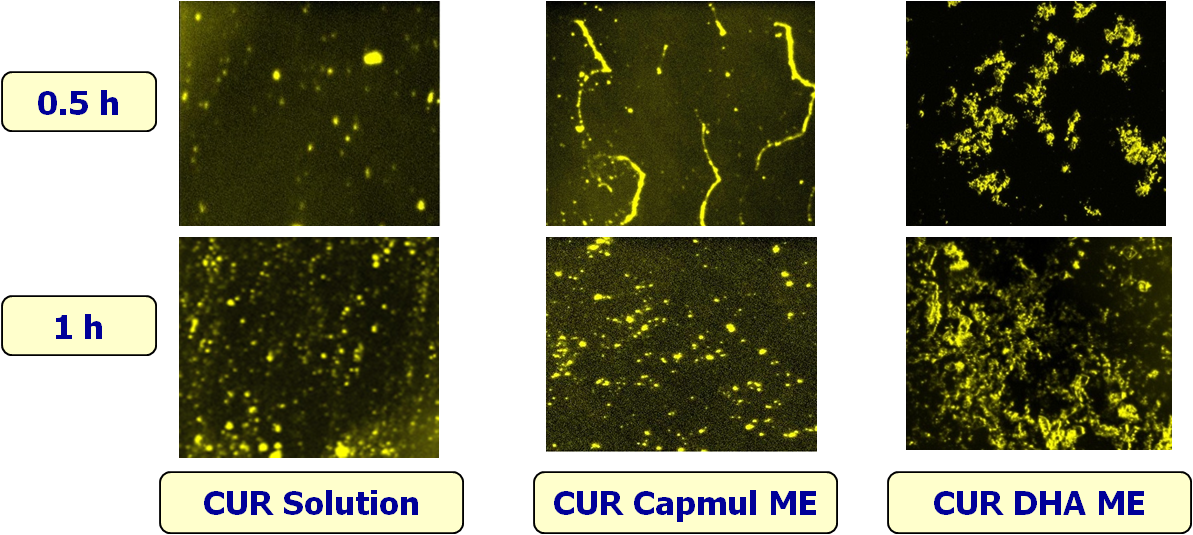


**Figure S2:** Fluorescence micrograph of brain sections at 0.5 and 1h

Explanation: Fluorescence images of brain tissue sections which showed significant yellow fluorescence confirmed CUR brain uptake, while the enhanced intensity of the yellow fluorescence established the superiority of the CUR DHA ME in enhancing brain uptake.


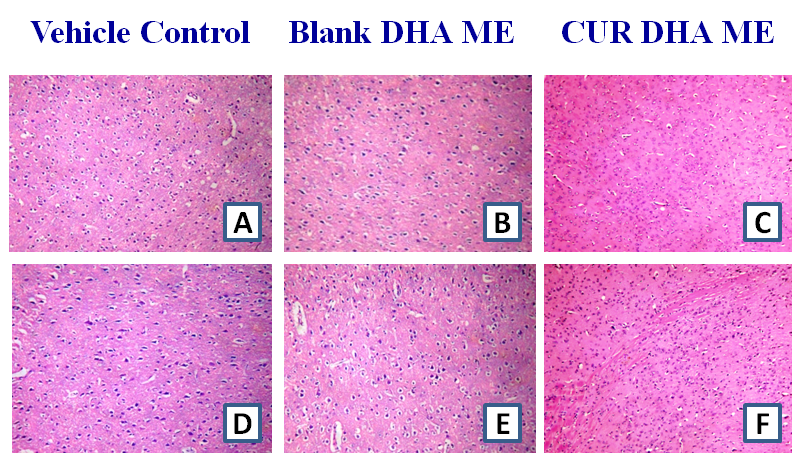


**Figure S3:** Photomicrographs of brain following intravenous administration Vehicle Control (A, 14 days), Blank DHA ME (B, 14 days)] and CUR DHA ME (C, 14 days) and photomicrograph of brain following intranasal administration Vehicle Control (D, 14 days), Blank DHA ME (E, 14 days) and CUR DHA ME (F, 14 days) (10× 10 magnification, n = 4).

Explanation: Histopathology of brain revealed healthy appearance and no significant changes at the end of 14 days.


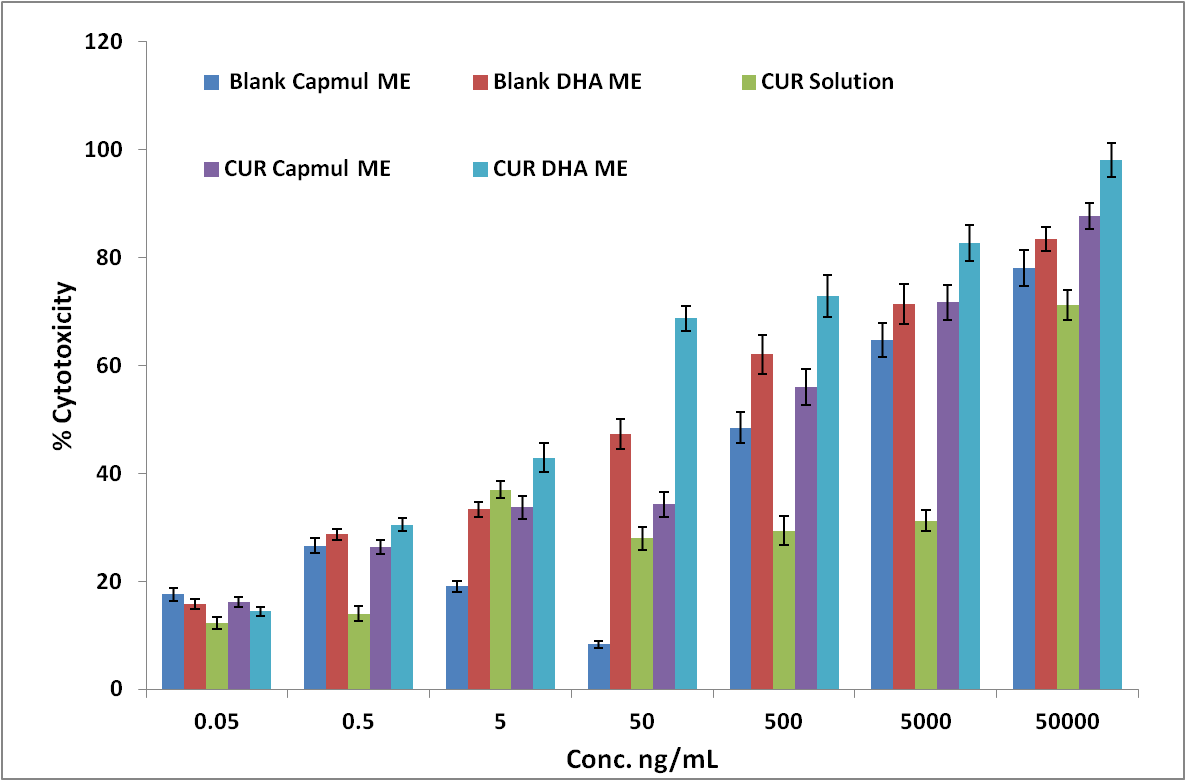


**Figure S4:** % Cell Cytotoxicity by MTT Assay at 1 h (mean ± S.D.; n = 3)

Explanation: Blank MEs were prepared corresponding to equivalent dilutions of CUR MEs. Blank Capmul ME revealed marginal cytotoxicity at lower dilution. The substantial increase in cytotoxicity at lower dilutions was attributed to possible surfactant based cytotoxicity. Interestingly the greater cytotoxicity exhibited by the blank DHA MEs compared to the similar CUR solution of similar dilution confirmed the anticancer property of DHA reported against glioblastoma cell line. Among the CUR MEs the CUR DHA ME revealed significantly higher cytotoxicity compared to the CUR Capmul ME ascribed to a possible synergy of DHA with CUR (Siddiqui et al. 2013a).
